# Innovations in kidney stone management: mini-PCNL for staghorn calculi in resource-limited settings

**DOI:** 10.3389/fruro.2025.1555624

**Published:** 2025-05-08

**Authors:** An Minh Nguyen, Hung Hai Do, Duc Van Nguyen, Long Hoang Vo

**Affiliations:** ^1^ Department of Surgery, Hanoi Medical College, Hanoi, Vietnam; ^2^ Department of Urology, Saint Paul General Hospital, Hanoi, Vietnam; ^3^ Department of Surgery, Thai Binh University of Medicine and Pharmacy, Thai Binh, Vietnam; ^4^ Department of Surgery, Hai Duong Hospital, Hai Duong, Vietnam; ^5^ Department of Science, Technology, Communication & International Cooperation, E Hospital, Hanoi, Vietnam

**Keywords:** mini-PCNL, staghorn calculi, holmium laser lithotripsy, resource-limited settings, kidney stone management

## Abstract

**Introduction:**

This study evaluates our initial experience with miniaturized percutaneous nephrolithotomy (mini-PCNL) in Vietnamese patients with staghorn calculi, using an 18F metal access sheath. This technique addresses the challenges of complex kidney stone management in resource-limited settings.

**Methods:**

A multi-center retrospective review of 236 patients with staghorn calculi who underwent mini-PCNL with high-power Ho laser lithotripsy (Lumenis 100 W) was conducted at four provincial hospitals in northern Vietnam from January 2020 to December 2023.

**Results:**

Among the 236 patients (mean age 54.88 years), 13.56% had prior open surgery, and 3.81% had previous PCNL. Presenting symptoms included flank/back pain (97.88%), acute renal colic (11.44%), and dysuria (5.93%). Right-sided stones were present in 55.93%, left-sided in 32.63%, and bilateral in 11.44%. The mean stone size was 28.05 mm, with 53.81% having stones of 20–30 mm, 38.56% over 30 mm, and 7.63% under 20 mm. Single stones were noted in 69.07%, while 30.93% had multiple stones. The mean stone surface area was 318.17 mm². Hydronephrosis was observed in 53.81% (grade-1: 32.64%; grade-2: 17.37%; grade-3: 3.81%). Postoperative complications included bleeding (13.14%), fever (9.75%), and reoperation or JJ stent placement (1.69%). Stone clearance rates were 67.37% at three days and 80.91% after one month. The mean durations for ureteral catheterization, postoperative hospitalization, and total hospital stay were 3.29, 6.94, and 12.90 days, respectively.

**Conclusions:**

Mini-PCNL with high-power Ho laser lithotripsy demonstrates safety and efficacy in managing staghorn calculi, achieving favorable stone clearance and recovery outcomes. This approach offers a viable, cost-effective solution for enhancing access to advanced urological care in resource-constrained environments.

## 1 Introduction

Various lithotripsy techniques, such as ultrasonic, electrohydraulic, and pneumatic methods, have been developed for the management of renal stones. Among these, the holmium (Ho) laser has recently emerged as a leading endoscopic lithotripter, demonstrating superior efficacy in achieving complete stone clearance, particularly in challenging cases like staghorn calculi. Over the years, percutaneous nephrolithotomy (PCNL) has evolved through numerous refinements in percutaneous access techniques, establishing itself as the gold standard for treating renal stones, especially those with a total stone burden greater than 20 mm (1, 2). For such large stones, PCNL is the first-line treatment recommended in current international guidelines, irrespective of the intrarenal stone location. For smaller stones with a burden of 20 mm or less, treatment options vary, including shock wave lithotripsy (SWL) or ureteroscopy (URS), depending on the stone’s location within the kidney (1, 2). For lower pole renal stones with a total burden of 10 mm or less, SWL or URS remains the recommended approach (1, 2).

Since its introduction in 1976, PCNL has evolved to enhance stone clearance and reduce complications, with one of the most notable innovations being the miniaturization of the access sheath. Standard PCNL typically employs a sheath size of 24 to 30 F, while miniaturized percutaneous nephrolithotomy (mini-PCNL), first introduced by Jackman (3) and later refined by Lahme (4), utilizes a sheath size of 14 to 20 F. Initially developed for 1–2 cm stones, mini-PCNL is now widely applied for all upper urinary tract stones larger than 1 cm, including both partial and complete staghorn stones.

Staghorn stones pose significant challenges for urologists, with a higher risk of perioperative complications compared to non-staghorn stones (5). Mini-PCNL offers the potential for more effective management with fewer complications. The increasing prevalence of renal stones, particularly staghorn calculi, poses a significant challenge to healthcare systems worldwide, particularly in resource-limited settings. Staghorn stones are known for their complexity and higher risk of complications during surgical intervention, necessitating advanced management strategies to ensure effective treatment while minimizing patient risk. With the advent of miniaturized techniques such as mini-PCNL, there is a pressing need to evaluate their applicability and effectiveness in diverse healthcare environments, especially where surgical resources and expertise may be limited.

In Vietnam, standard PCNL was first introduced in 1997, with mini-PCNL using 16–20 Fr tracts adopted by 2008. Studies have demonstrated mini-PCNL’s comparable efficacy to standard PCNL, along with reduced complications and shorter hospital stays (6–8). Achieving optimal stone-free rates (SFR) is contingent on several factors, particularly stone burden (9), but even large and complex stone burdens can now be managed effectively with mini-PCNL.

However, the resource constraints faced by many provincial hospitals in Vietnam may limit the ability to fully implement advanced techniques like mini-PCNL. Thus, it is crucial to evaluate its feasibility and outcomes in settings with limited resources. This study is critical as it not only seeks to explore the efficacy of mini-PCNL with high-power holmium laser lithotripsy but also aims to provide insights into its practical implementation in provincial hospitals. Specifically, this multi-center study was conducted across four provincial hospitals in the northern region of Vietnam, allowing a comprehensive assessment of its applicability and effectiveness in real-world, resource-constrained settings. This report presents our initial experience with mini-PCNL using high-power Ho laser lithotripsy for staghorn calculi and discusses the implications for its clinical application in resource-scarce environments. Our findings will contribute to future studies comparing the efficacy of mini-PCNL with standard PCNL for staghorn stones, particularly in Vietnam.

## 2 Materials and methods

### 2.1 Study design and patients

This retrospective study involved 236 patients with staghorn calculi, defined as branched stones occupying the renal collecting system, who underwent mini-PCNL using a high-pulsed Ho laser (Lumenis 100 W) at four provincial hospitals in the northern region of Vietnam between January 2020 and December 2023. Exclusion criteria included severe urinary tract infections, uncontrolled diabetes, bleeding disorders, pregnancy, abdominal aortic aneurysm, renal artery stenosis/aneurysm, congenital anomalies of the kidney and urinary tract, ureteral strictures, and ureteropelvic junction obstruction. Clinical profiles, preoperative characteristics, intraoperative details, and postoperative outcomes were collected from the patient’s medical records and the hospital’s computerized database.

All procedures conducted in this study adhered to the ethical standards of the Institutional and National Research Committees, as well as the 1964 Helsinki Declaration and its subsequent amendments or comparable ethical standards. Informed consent was obtained from all patients prior to surgery, and they agreed to the use of their medical data for clinical research purposes.

### 2.2 Research instruments

The study utilized various imaging and surgical instruments. For imaging, a 300W Xenon cold light source, a PAL color camera with a resolution of 450 lines and zoom capability (25–50 mm), a high-resolution monitor designed for endoscopic surgery, a Philips ultrasound machine with a 3.5 MHz probe, a Senda QB-1 water pump, and a Karl Storz semi-rigid endoscope (13.5 F) were employed (Image 1: Karl Storz semi-rigid endoscope, 13.5 F). Surgical instruments included a standard operating table with adjustable positions, a Dosantos 18G x 220 mm puncture needle, a scalpel with a sharp blade, (Image 2: Needle puncture and dilatation instruments for renal tunneling) a Lunderquist Guidewire, a 6Fr Guidewire for ureteral catheter placement, an Amplazt plastic dilation set (18F), a 100W laser lithotripsy machine (Lumenis^®^ Pulse™ P120H, Lumenis Ltd., Yokneam, Israel), and an irrigation system connected to the ureteral catheter (NQ). Isotonic saline solution (0.9%) was used for irrigation.

### 2.3 Surgical procedure

#### 2.3.1 Stage 1: preparation for percutaneous nephrolithotomy

The patient was administered either spinal or general anesthesia and placed in the lithotomy position. Ureteral catheterization was performed to access the upper urinary tract, facilitating the identification of any urinary tract abnormalities and ensuring ureteral patency. Continuous saline infusion under controlled hydrostatic pressure (5–20 kilopascals) was utilized to distend the renal collecting system, optimizing conditions for percutaneous access, dilation, and subsequent stone fragmentation. After the ureteral catheter was placed, a Foley catheter was introduced to maintain bladder drainage, and the ureteral catheter was secured alongside the Foley catheter to prevent displacement during the procedure. The ureteral catheter was then connected to an irrigation system to maintain consistent fluid flow during the surgery. Subsequently, the patient was repositioned into the prone position to facilitate renal access. The primary surgeon stood on the ipsilateral side of the kidney to be treated, while two assistant surgeons were positioned contralaterally. Laparoscopic and ultrasound monitors were appropriately aligned with the surgeon’s field of vision to ensure real-time visualization during the procedure.

#### 2.3.2 Stage 2: performing percutaneous nephrolithotomy

##### 2.3.2.1 Renal calyx puncture

The initial step involved ultrasound-guided puncture of the targeted renal calyx, which had been preoperatively identified on computed tomography (CT) imaging. Serial dilation of the nephrostomy tract was performed up to 18 French (Fr) using an Amplatz sheath under continuous ultrasound guidance to ensure accurate access to the renal pelvis. An 18 Fr plastic dilator was advanced through the Amplatz sheath until the tip reached the renal pelvis, completing the nephrostomy tract formation. The Amplatz sheath was carefully advanced, and the dilator was gradually withdrawn, confirming the correct placement of the sheath within the renal pelvis. Throughout this phase, bleeding was continuously assessed using the Modified Satava Classification System, with minor bleeding managed by irrigation and temporary tamponade, while more significant bleeding addressed with electrocautery as needed. This puncture and dilation phase represented the most technically complex aspect of the procedure.

##### 2.3.2.2 Laser lithotripsy

Once the nephrostomy tract was established, the Holmium laser lithotripsy system (100-watt) was employed for stone disintegration. The Amplatz sheath was maneuvered into optimal position near the renal calculus, and laser lithotripsy was performed to fragment the stone into smaller pieces. The assistant surgeon stabilized the Amplatz sheath during the lithotripsy process to prevent inadvertent movement. Stone fragments were extracted using forceps and a dormia basket. Following the completion of lithotripsy and stone extraction, a thorough inspection of the renal collecting system was performed to ensure no residual calculi remained. A double-J (JJ) ureteral stent was then placed to ensure postoperative drainage and prevent obstruction, and a 12–14 Fr Foley catheter was introduced into the nephrostomy tract via the Amplatz sheath. The balloon of the Foley catheter was inflated with 3 mL of sterile saline to secure its position, and the Amplatz sheath was subsequently removed. The nephrostomy tract was then sealed, and the external drainage system was appropriately secured to the patient’s skin.

### 2.4 Variables and definitions

In this analysis, several key variables were assessed to evaluate the outcomes of miniaturized PCNL with Holmium laser lithotripsy in patients with staghorn calculus. Preoperative characteristics included patient demographics (age, gender), body mass index (BMI), previous urologic treatments, and symptoms (e.g., flank pain, renal colic). Imaging variables encompassed stone location (right kidney, left kidney, or both), stone size (measured in millimeters), and stone burden classified as small (<20 *mm*), intermediate (20*–*30 mm), or large (>30 *mm*). All preoperative and postoperative CT imaging was performed using standardized 64-slice scanners (Philips Brilliance) with consistent parameters: 2*–*3 mm slice thickness, 120 kVp tube voltage, and automated dose modulation.

Intraoperative variables included the number of punctures, the type and number of percutaneous tracts used, the duration of percutaneous puncture, lithotripsy, and total operative time. Postoperative bleeding complications were systematically categorized and quantified using standardized criteria: (1) Minor bleeding was defined as hemoglobin drop <2 g/dL without transfusion requirement; (2) Major bleeding included hemoglobin drop ≥2 g/dL or transfusion requirement; and (3) Severe bleeding encompassed cases requiring angioembolization or surgical reintervention. Comprehensive bleeding assessment protocols were implemented, including serial hemoglobin measurements (preoperatively, at 6 hours postoperatively, and daily until discharge), intensive vital sign monitoring (every 2 hours for the initial 24 hours), and meticulous documentation of all transfusion events. Postoperative characteristics comprised complications (graded bleeding as above, fever, re-operation), pain management (duration and type of analgesics used), and stone-free status at 3 days and 1 month post-procedure. Laboratory values assessed included RBC count, hematocrit, hemoglobin, WBC count, BUN, and creatinine levels.

Stone-free status was defined as the absence of residual stones or the presence of fragments ≤4 mm, as confirmed by non-contrast CT scans performed three days and one month postoperatively. All postoperative CT scans were independently reviewed by blinded radiologists using standardized measurement tools, with discrepancies resolved by consensus review with a senior radiologist. Fragments ≤4 mm were considered clinically insignificant based on their low likelihood of causing symptoms or requiring further intervention. CT scans were chosen due to their high sensitivity and specificity in detecting even small residual fragments, ensuring accurate assessment of stone-free status.

For comprehensive evaluation of treatment success, surgical outcomes were categorized into three groups: (1) Good outcome - defined as complete stone-free status (no fragments or fragments ≤4 mm) without any major complications (no transfusion-requiring bleeding, febrile UTI, or re-intervention); (2) Average outcome - presence of residual fragments (4-10mm) and/or minor complications (transient fever managed medically, minor bleeding with hemoglobin drop <2g/dL); and (3) Poor outcome - significant residual stones (>10mm) and/or severe complications (sepsis, angioembolization, or conversion to open surgery). This classification system was developed to provide clinically meaningful endpoints that reflect both therapeutic efficacy and patient safety, particularly relevant in resource-constrained settings where optimal outcomes must balance stone clearance with procedural morbidity.

### 2.5 Data analysis

An initial quality control assessment was performed to identify potential coding errors, outliers, or non-standard/non-normal distributions in the dataset. All statistical analyses were performed with Stata^®^ 15 (StataCorp LLC, USA). Main descriptive statistics were reported as absolute and relative (%) frequencies for categorical variables or as means with their standard deviation (SD) or median and interquartile range (IQR), depending on the normality of the distribution.

## 3 Results

### 3.1 Preoperative characteristics

A total of 236 patients were retrospectively analyzed in this study. The demographic and clinical characteristics of the cohort are summarized in **Table 1**. The mean age of the patients was 54.88 years (± 11.93, range: 30–80 years). The majority of patients were male (59.75%), with the predominant occupational category being blue-collar workers (92.37%). A small proportion of the patients were underweight (11.44%), and the rest had normal weight (88.56%). A history of prior urological interventions was noted in 13.56% of patients who had undergone open surgery, and 3.81% had previously received PCNL.

**Table 1:** Preoperative characteristics of the study population.

Preoperative variables	All Patients (N = 236)	
	Count	% of total
Gender - %		
Male	141	59.75
Female	95	40.25
Age group - %		
≤30 years	5	2.12
31–50 years	82	34.75
51–70 years	132	55.93
>70 years	17	7.20
Occupation - %		
Blue-collar worker	218	92.37
White-collar worker	5	2.12
Freelancer	13	5.51
BMI classification - %		
Underweight	27	11.44
Normal weight	209	88.56
Previous urologic treatment - %		
No	195	82.63
Percutaneous nephrolithotomy	9	3.81
Open surgery	32	13.56
Clinical symptoms on admission		
Acute renal colic - %	27	11.44
Pain in the flanks and back - %	231	97.88
Dysuria - %	14	5.93
Side of kidney stones - %		
Right kidney	132	55.93
Left kidney	77	32.63
Both kidneys	27	11.44
Size of kidney stones - %		
< 20 mm	18	7.63
20–30 mm	127	53.81
> 30 mm	91	38.56
Number of kidney stones -%		
1 stone	163	69.07
2 stone	18	7.63
≥3 stone	55	23.31
Stone surface area - %		
100 mm^2^ - < 200mm^2^	54	22.88
200 mm^2 -^ < 300mm^2^	68	28.81
300 mm^2^	114	48.31
Staghorn classification - %		
Complete staghorn	9	3.81
Partial staghorn	227	96.19
Degree of hydronephrosis ^a^ - %		
Grade 0	109	46.19
Grade 1	77	32.64
Grade 2	41	17.37
Grade 3	9	3.81
Kidney failure - %		
No	213	90.25
Yes	23	9.74
Urinary erythrocytes -%		
Negative	145	61.44
Positive	91	38.56
Urinary leukocytes -%		
Positive	141	59.74
Negative	95	40.25
	Mean ± SD	IQR
Patient age (years)	54.88 ± 11.93	30 – 80
Size of kidney stones (mm)	28.05 ± 5.58	17 – 39
Stone surface area (mm^2^)	318.17 ± 127.74	106 - 588.8
RBC (T/l)	4.84 ± 0.71	3.7 – 5.7
Hematocrit (%)	43.08 ± 4.23	34.1 – 52
Hemoglobin (g/l)	141.39 ± 15.11	110.8 – 175
WBC	8.07 ± 2.36	4.4 – 15.6
Platelet count	278.72 ± 73.31	125.4 – 464.4
BUN (mmol/L)	5.69 ± 1.61	2.4 – 11.0
Creatinine (μmol/L)	88.1 ± 21.8	50 – 137

a Hydronephrosis was graded into four degrees according to Beetz et al

RBC, red blood cells; WBC, white blood cells; BUN, Blood urea nitrogen

SD, standard deviation; IQR, interquartile range

Regarding symptomatology, flank and back pain were the most commonly reported symptoms, affecting 97.88% of the cohort. Acute renal colic was present in 11.44% of patients, while dysuria was reported in 5.93%. Imaging studies demonstrated that 55.93% of patients had staghorn stones in the right kidney, 32.63% in the left kidney, and 11.44% had bilateral stones. The average stone size was 28.05 mm (± 5.58, range: 17–39 mm). Intermediate-sized stones (20–30 mm) were the most frequently encountered (53.81%), followed by stones larger than 30 mm (38.56%), and stones smaller than 20 mm (7.63%). Most patients (69.07%) had a solitary stone, while 30.93% had multiple renal stones. The mean stone surface area was 318.17 mm² (± 127.74, range: 106–588.8 mm²) (**Table 1**).

Hydronephrosis was identified in 53.81% of patients, with 32.64% presenting with grade-1 hydronephrosis, 17.37% with grade-2, and 3.81% with grade-3. Nearly half of the patients (46.19%) exhibited no evidence of renal pelvis dilatation on imaging (**Table 1**).

Preoperative laboratory assessments indicated a mean red blood cell count (RBC) of 4.84 T/L, hematocrit levels of 43.08%, hemoglobin concentrations of 141.39 g/L, a white blood cell (WBC) count of 8.07 × 10^9^/L, and a platelet count of 278.72 × 10^9^/L. The mean blood urea nitrogen (BUN) and serum creatinine levels were 5.69 mmol/L and 88.1 μmol/L, respectively. Normal renal function was observed in 90.25% of patients, with 9.74% exhibiting signs of renal insufficiency. Notably, erythrocytosis was present in 38.56% of the cohort, and leukocytosis was detected in 59.74% of patients (**Table 1**).

### 3.2 Intraoperative and postoperative characteristics

**Table 2** summarizes the intraoperative events and outcomes. Of the patients, 84.74% required only one puncture into the renal pelvis, 11.44% required two punctures, and 3.81% required three punctures. Among the patients, 71.19% underwent the procedure using a single percutaneous tract, while 28.81% had two tracts. The positions for puncture included the middle calyx in 59.74% of patients, the lower calyx in 36.44%, and the upper calyx in 3.81%. Intraoperative parameters recorded included a percutaneous puncture duration of 12.31 minutes (± 6.14, range 5–30), a lithotripsy duration of 57.94 minutes (± 17.00, range 35–100), and a total operative duration of 82.46 minutes (± 21.83, range 52–135).

**Table 2:** Intraoperative and postoperative characteristics.

Operative variables	All Patients (N = 236)	
	Count	% of total
Percutaneous puncture of renal pelvis - %		
1 time	200	84.74
2 times	27	11.44
3 times	9	3.81
Number of tracts - %		
1 tract	168	71.19
2 tracts	68	28.81
Positions for puncture - %		
Upper calyx	9	3.81
Middle calyx	141	59.74
Lower calyx	86	36.44
Postoperative variables		
Bleeding - %	31	13.14
Fever - %	23	9.75
Re-operation & JJ stent placement - %	4	1.69
Pain medications after surgery - %	86	36.44
Ureteral catheter circulation time - %		
3–5 days	218	92.37
> 5 days	18	7.63
Stone free status three days after surgery - %		
Yes	159	67.37
No	77	32.63
Surgical outcome - %		
Good	159	67.37
Average	73	30.93
Poor	4	1.69
Stone free status one month after surgery - %		
Yes	191	80.91
No	45	19.09
	Mean ± SD	IQR
Operative variables		
Percutaneous puncture duration (minutes)	12.31 ± 6.14	5 – 30
Lithotripsy duration (minutes)	57.94 ± 17.o0	35 – 100
Total operative duration (minutes)	82.46 ± 21.83	52 – 135
Postoperative variables		
RBC (T/l)	4.53 ± 0.51	3.15 – 5.8
Hematocrit (%)	38.89 ± 3.99	28.7 – 47
Hemoglobin (g/l)	132.01 ± 16.73	97 – 191.2
WBC	8.43 ± 1.42	5 – 14.4
BUN (mmol/L)	5.21 ± 1.21	4.2 – 9.1
Creatinine (μmol/L)	83.80 ± 17.41	62 – 127
Ureteral catheter circulation time (days)	3.29 ± 1.52	3 – 10
Length of postoperative hospitalization (days)	6.94 ± 2.41	5 – 15
Total hospital stay (days)	12.90 ± 6.39	7 – 28

RBC, red blood cells; WBC, white blood cells; BUN, Blood urea nitrogen

SD, standard deviation; IQR, interquartile range

As shown in **Table 2**, postoperative events included bleeding in 13.14% of patients (31/236) - categorized as minor (8.05%; 19/236), major (4.24%; 10/236), and severe (0.85%; 2/236 requiring reoperation) - along with fever in 9.75% (23/236) and elective re-operation with JJ stent placement in 1.69% (4/236). Thirty-six point four percent (36.44%) of patients required pain medications after mini-PCNL. Postoperative main laboratory findings revealed that the mean RBC, hematocrit, hemoglobin, WBC, BUN, and creatinine levels were 4.53 T/L, 38.89%, 132.01 g/L, 8.43 × 10^9^/L, 5.21 mmol/L, and 83.80 μmol/L, respectively.

Three days after mini-PCNL, a stone-free status was achieved in 67.37% of patients. One month after the procedure, the stone clearance rate was 80.91%. The mean duration of ureteral catheter circulation was 3.29 days (± 1.52, range 3–10), the mean length of postoperative hospitalization was 6.94 days (± 2.41, range 5–15), and the mean total hospital stay was 12.90 days (± 6.39, range 7–28). Overall, a favorable surgical outcome was recorded for the entire cohort, with 67.37% of patients achieving a good surgical outcome, 30.93% achieving an average outcome, and 1.69% having a poor outcome (**Table 2**).

## 4 Discussion

Our study demonstrates that mini-PCNL is feasible for staghorn calculi in Vietnamese provincial hospitals despite three key constraints: (i) diagnostic delays (mean stone size 28.05mm), (ii) equipment limitations (ultrasound-guided access), and (iii) financial barriers (92% blue-collar patients). Kidney stone disease is a prevalent urologic disorder globally, and Vietnam is no exception. Patients often present with advanced cases, which complicates treatment and outcomes.

In our study, 55.93% of patients had staghorn stones in the right kidney, and 11.44% had bilateral stones, reflecting the complexity of cases treated. Furthermore, limited insurance coverage and the high cost of treatment add significant financial strain on patients and their families. This financial burden restricts access to timely, effective care, especially in rural and under-resourced areas. Existing literature on the management of kidney stone disease predominantly comes from high-resource settings and large institutions in developed countries, where the availability of advanced technology and well-established healthcare infrastructures support favorable outcomes. However, little data is available regarding the application of mini-PCNL in lower-resource environments such as provincial hospitals in Vietnam. Specifically, the literature lacks insight into the safety, efficiency, and postoperative outcomes of mini-PCNL for complex cases like staghorn calculi under these challenging conditions. With advancements in technology, the miniaturization of access sheaths has revolutionized the field of PCNL. Modern techniques have refined PCNL into several variants, including mini-PCNL (≤22 Fr), Chinese mini-PCNL (14–20 Fr), super-mini-PCNL (10–14 Fr), ultra-mini-PCNL (11–13 Fr), micro-PCNL (4.8 Fr), and mini-micro-PCNL (8 Fr) (10). Mini-PCNL, which utilizes smaller-diameter instruments and access sheaths, has shown promise in reducing complications and recovery time. Numerous authors have detailed the utilization of various endoscopes for the fragmentation and extraction of stones, with access sheath sizes ranging from 11 to 20 Fr (11–13). In our study, we utilized the 18F mini-nephroscope with an Amplatz sheath, disintegrating stones with a holmium laser, a technique highly regarded for its ability to fragment stones into small, dust-like particles. However, challenges remain, such as the gap between the inner part of the Amplatz sheath and the mini-nephroscope, which is critical for flushing out stone fragments but can pose technical difficulties, particularly in resource-constrained environments like ours.

Operative duration is a critical factor in assessing the advantages of any surgical technique. One of the noteworthy benefits of mini-PCNL, which was serendipitously discovered, is the automatic expulsion of small stone fragments from the calyceal system due to irrigation backflow. This phenomenon occurs as a result of the specific dimensions of the Amplatz sheath, which facilitates passive stone clearance and reduces the need for manual extraction. This feature contributes to operating times that are comparable to those of conventional PCNL, despite the smaller sheath size. According to Bikash Bikram Thapa (9), while mini-PCNL may involve slightly longer operative times than standard PCNL due to the sheath size and the process of retrieving stone fragments, studies such as Zhong (14) have demonstrated that mini-PCNL significantly reduces overall operative time. Similarly, both Du (15) and Zhu (16) reported shorter stone clearance times in mini-PCNL procedures compared to traditional PCNL, underscoring its efficiency. Reduced operative time is a crucial goal as it minimizes the risk of surgical, anesthesia, and postoperative complications, while also lowering healthcare costs, an essential consideration in resource-limited settings. In this study, the average total operative time was 82.46 minutes, with an average percutaneous puncture duration of 12.31 minutes and an average lithotripsy duration of 57.94 minutes. These findings reflect the efficiency of mini-PCNL in the practice conditions of provincial hospitals in Vietnam and are consistent with other studies highlighting its time-saving benefits.

The findings of this study demonstrate a relatively high stone clearance rate and an acceptable complication profile for the mini-PCNL procedure in Vietnamese patients. Notably, advancements in surgical techniques and technology have significantly minimized the risk of complications in PCNL when performed by experienced hands. Our results showed that the SFR was 67.37% at three days postoperatively and improved to 80.91% after one month, which aligns with previous studies emphasizing the efficacy of mini-PCNL in managing complex stones. Postoperative complications were observed, including bleeding in 13.14% of patients, fever in 9.75%, and 1.69% requiring reoperation for JJ stent placement, reflecting an acceptable safety profile. The use of an 18F Amplatz sheath in mini-PCNL, much smaller than the 30F sheath used in conventional PCNL, reduces the cross-sectional area to only 20% of that in standard procedures. This reduction is critical in decreasing the risk of bleeding and the need for blood transfusions, a well-documented advantage of mini-PCNL over traditional PCNL. In our study, no patient required conversion to open surgery, and all bleeding cases were managed conservatively. Similar findings were reported by Truong Pham Ngoc Dang (17), where intraoperative bleeding led to the cessation of surgery in three patients, two of whom required blood transfusions postoperatively. These cases were associated with longer operative times (120 minutes and 135 minutes). In our cohort, the mean operative duration was 82.46 minutes, with the majority of cases requiring only a single puncture (84.74%), which likely contributed to the lower complication rates compared to reports with more complex cases. As higher SFR are often correlated with an increased risk of complications, particularly bleeding and transfusions (18), techniques like mini-PCNL have been developed to minimize parenchymal trauma by employing smaller access sheaths and nephroscopes, thereby reducing the risk of bleeding. In this study, the average duration of ureteral catheter circulation was 3.29 days, and the mean postoperative hospitalization was 6.94 days, indicating that mini-PCNL not only achieves favorable SFRs but also supports a relatively quick recovery for patients.

Interestingly, our results align with those of Mohammed S. ElSheemy’s study (7), which also noted that patients with a history of previous open renal stone surgery were more likely to undergo mini-PCNL. In this study, 13.56% of patients had a history of prior open surgery, and 3.81% had previously undergone PCNL. Despite these prior interventions, mini-PCNL demonstrated favorable outcomes, including a low complication rate (bleeding: 13.14%; fever: 9.75%; re-operation: 1.69%) and a high SFR of 80.91% at one month postoperatively. These findings highlight the safety and efficacy of mini-PCNL, even in patients with complex surgical histories. This highlights the potential of mini-PCNL as a safer alternative, particularly in complex cases, while still maintaining high efficacy in stone clearance. These findings reinforce the importance of continued refinement in surgical techniques and the adaptation of mini-PCNL to resource-constrained settings like those in provincial hospitals in Vietnam, where technological limitations often necessitate the use of smaller, less invasive instruments.

At our institution, stone clearance was defined as the absence of stones or the presence of stone fragments ≤4 mm, as confirmed by postoperative C-arm imaging and urinary system radiographs within the first three days following surgery. While mini-PCNL showed a favorable complication profile, concerns remain about its relative efficacy in clearing large stones, as the smaller tract may limit the use of instruments needed for larger calculi. In conventional PCNL, large stone fragments often require active removal using forceps or baskets, whereas mini-PCNL relies on irrigation flow to wash out fragments, minimizing the need for these tools. This method represents a key distinction, allowing for the treatment of even large stones within an acceptable operative time. In our case series, we reported a 67.37% SFR three days postoperatively and an 80.91% SFR one month postoperatively, aligning with reported outcomes from similar procedures in resource-limited settings (18, 19). Given the retrospective nature of our study, SFRs were analyzed only at three days and one month post-surgery, reflecting the limitations of institutional data. However, the stone clearance rate in our cohort was still considered acceptable.

Several retrospective studies have also demonstrated the safety and efficacy of mini-PCNL, encouraging broader adoption of the technique for large, complex, and staghorn stones (6, 20, 21). For example, Guohua (22) achieved a 93% SFR for staghorn stones using mini-PCNL, while Zhao (23) reported an 84% SFR in two-stage, multi-tract mini-PCNL. Similarly, Hu G.’s study on 1,368 patients showed an 82% SFR with a 16F tract (24), Ozgor F. reported an 80.4% SFR with a mean stone size of 19.5 mm (25), and Knoll T. reported a 96% SFR with a mean stone size of 18 mm (26). Kirac, in his study, achieved a 91.9% SFR with a mean stone size of 10.5 mm (27). Our SFR was 67.37% at three days and 80.91% at one month postoperatively, which is in line with results from other studies in resource-limited settings, though slightly lower due to the larger stone sizes in our patient cohort. Nevertheless, previous studies have consistently highlighted mini-PCNL as a reliable option for treating larger upper urinary tract stones, offering comparable outcomes to conventional PCNL but with significantly lower morbidity (28). Importantly, mini-PCNL is not just a scaled-down version of conventional PCNL but a fundamentally distinct approach, with differences in instrument size, lithotripsy techniques, stone removal methods, and complication profiles.

A new surgical approach in this study highlights the innovation embodied by mini-PCNL for managing complex kidney stones in our clinical context. Mini-PCNL utilizes smaller access tracts (≤22 Fr), resulting in reduced trauma to surrounding tissues and minimized postoperative complications such as bleeding and tissue damage, while maintaining efficacy in stone clearance. This approach is particularly vital in environments where advanced healthcare resources are limited, making traditional techniques less feasible. The adaptability of Mini-PCNL to low-resource settings allows it to be performed with fewer specialized instruments, reducing the demand for blood transfusions and intensive postoperative care, which is often unavailable in provincial hospitals. The findings of this study demonstrate that mini-PCNL achieves SFR comparable to conventional PCNL, even in challenging cases like large stones and staghorn calculi. Additionally, mini-PCNL significantly lowers the incidence of perioperative complications, including the risk of bleeding and adjacent organ injury. By integrating advanced technology in a manner that is accessible to hospitals with limited infrastructure, mini-PCNL not only provides a safe and effective treatment alternative but also enhances patient outcomes in regions with restricted access to high-end surgical care. Thus, the introduction of mini-PCNL represents a significant advancement in the surgical management of complex kidney stones in Vietnam. In addition, this study’s multicenter design, involving data from four provincial hospitals, strengthens the reliability and applicability of the findings across different settings. The involvement of multiple centers enhances the generalizability of the results, demonstrating that mini-PCNL is a viable and effective approach for managing complex kidney stones, even in resource-constrained environments.

The management of residual stones in our cohort primarily involved metabolic evaluation (e.g., 24-hour urine analysis) and scheduled imaging follow-ups at 3-month intervals, particularly for fragments >4mm. This approach aligns with protocols in resource-constrained settings where secondary procedures carry significant financial burdens. Notably, 36.44% of patients required postoperative analgesia, predominantly due to transient renal capsule irritation from tract dilation or residual micro-fragments. Most cases were managed effectively with non-opioid analgesics (e.g., paracetamol/NSAIDs), underscoring the technique’s minimally invasive nature. Future studies should standardize pain assessment tools to further optimize perioperative care.

Our study has several limitations, primarily stemming from its retrospective design, which inherently restricts the ability to control for potential confounding factors. Additionally, while the sample size of 236 patients is relatively adequate, the short follow-up period may limit the generalizability of our findings. Another notable limitation is the absence of a control group undergoing conventional PCNL, which would have allowed for direct comparison of efficacy and safety outcomes between mini-PCNL and the standard procedure. This was due to the predefined scope of our research objectives, which focused exclusively on mini-PCNL. However, despite the lack of a control group, several studies in the literature have compared mini-PCNL with conventional PCNL and reported comparable outcomes. To provide a more comprehensive understanding of the role of mini-PCNL, especially in the treatment of complex cases such as staghorn stones, future studies will need to address these limitations. Specifically, a larger cohort and a prospective, randomized design with a control group would significantly enhance the robustness and reliability of the evidence. This will enable a more thorough comparative analysis of the efficacy and safety profiles of mini-PCNL versus conventional PCNL, contributing to the broader body of knowledge on optimal surgical approaches for large renal stones.

## 5 Conclusions

Our study underscores the viability and effectiveness of mini-PCNL as a surgical option for the management of kidney stones, particularly in resource-limited settings such as provincial hospitals in Vietnam. The technique demonstrates favorable outcomes, including a high stone clearance rate and an acceptable complication profile, even in complex cases such as staghorn calculi. By utilizing smaller instruments and access sheaths, mini-PCNL significantly reduces operative duration and minimizes parenchymal trauma, thereby lowering the risk of complications typically associated with traditional PCNL. These findings advocate for the broader adoption of mini-PCNL in comparable healthcare settings, as it offers a promising alternative to conventional techniques, improving patient access to safe and effective urological care. Future research, particularly prospective randomized studies with larger cohorts, is essential to validate these results and further elucidate the role of mini-PCNL in the surgical management of kidney stones.

## Data Availability

The raw data supporting the conclusions of this article will be made available by the authors, without undue reservation.
